# Neonatal repair of total anomalous pulmonary venous connection accompanied by unilateral lung agenesis and Goldenhar syndrome: a case report

**DOI:** 10.1186/s13019-021-01722-5

**Published:** 2021-11-21

**Authors:** Takahiro Ito, Ikuo Hagino, Mitsuru Aoki, Kentaro Umezu, Tomohiro Saito, Akiyo Suzuki

**Affiliations:** Department of Cardiovascular Surgrey, Chiba Children’s Hospital, 579-1 Heta, Midori, Chiba-city, Chiba, 266-0007 Japan

**Keywords:** Total anomalous pulmonary venous connection, Goldenhar syndrome, Unilateral lung agenesis

## Abstract

**Background:**

Total anomalous pulmonary venous connection accompanied by unilateral lung agenesis and Goldenhar syndrome is extremely rare.

**Case presentation:**

We present a case of total anomalous pulmonary venous connection accompanied by unilateral lung agenesis and Goldenhar syndrome in a patient who was diagnosed based on transthoracic echocardiography and computed tomography. We observed complete absence of the lung, the bronchial tree, and vascular structures on the right side, with abnormal drainage of the left pulmonary veins into the innominate vein. The patient showed clear clinical evidence of pulmonary venous obstruction and underwent surgery 3 days after birth. The pulmonary venous chamber containing the vertical vein was anastomosed to the left atrium using 7–0 PDS running sutures via a median sternotomy. Echocardiography and computed tomography performed 1 year postoperatively revealed no pulmonary venous obstruction.

**Conclusion:**

We report a rare case of total anomalous pulmonary venous connection accompanied by unilateral lung agenesis and Goldenhar syndrome, which was successfully repaired 3 days after birth. A median sternotomy is a safe and effective approach for surgical repair of congenital heart disease with unilateral lung agenesis. Repair of the supra cardiac total anomalous pulmonary connection using the vertical vein is feasible in patients with a small pulmonary venous chamber.

## Introduction

Goldenhar syndrome is a rare congenital disease characterized by craniofacial abnormalities, including incomplete development of the eyes, ears, and jaw [1]. The mean incidence rate of this condition is estimated to be between 1:3000 and 1:5000 live births. Cardiovascular malformations are reported in 5–58% of patients [1], and the prevalence of lung agenesis is known to be 34 per 1,000,000 live births [2]. Previous studies have reported repair of total anomalous pulmonary venous connection associated with lung agenesis; however, the operative mortality was high in such cases. Moreover, only 2 case reports in the literature have described total anomalous pulmonary venous connection concomitant with unilateral lung agenesis and Goldenhar syndrome. We report a rare case of Goldenhar syndrome in a patient with unilateral lung agenesis and concomitant total anomalous pulmonary venous connection, which was successfully repaired 3 days after birth. To our knowledge, this is the first report that describes this rare condition in a patient who survived more than 1 year postoperatively.

## Case presentation

A 25-year-old pregnant woman was referred at 25 weeks’ gestation for fetal diagnosis of right lung agenesis and right eye hypoplasia, without clear evidence of cardiac malformations.

**Fig. 1:**
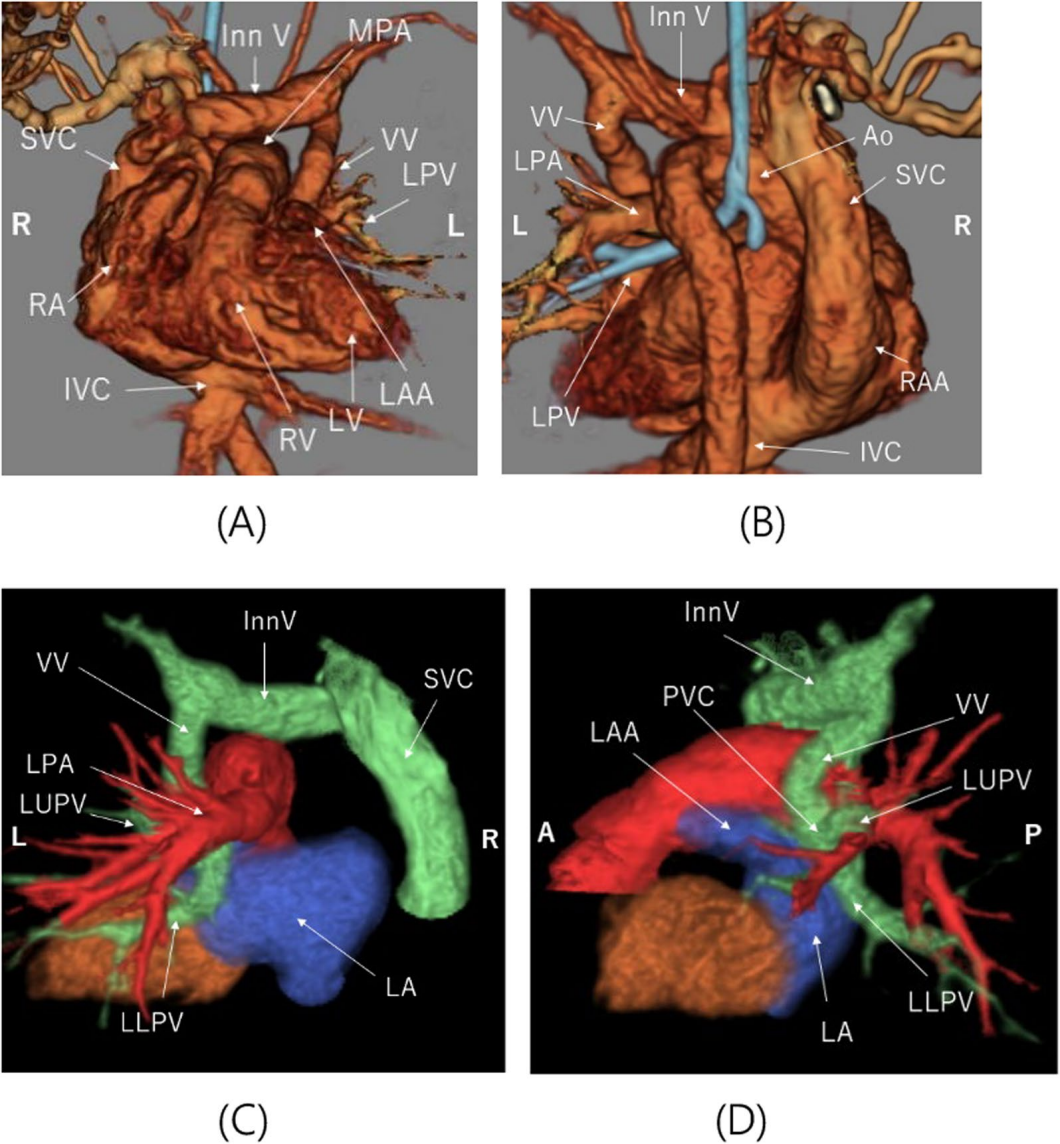
Preoperative 3-dimensional tomography. **A**, **B** Total absence of the right bronchial tree and right vascular structures (anterior to posterior view and posterior to anterior view). **C**, **D** Abnormal drainage of the left pulmonary veins into the innominate vein (posterior to anterior view and left side to right side view). LPV, left pulmonary vein; LUPV, left upper pulmonary vein; LLPV, left lower pulmonary vein; LPA, left pulmonary artery; VV, vertical vein; InnV, innominate vein; SVC, supra vena cava; Ao, aorta; IVC, inferior vena cava; RA, right atrium; LA, left atrium; RV, right ventricle; LV, left ventricle; LAA, left atrial appendage; PVC, pulmonary venous chamber

A female neonate born at 37 weeks via cesarean delivery (birth weight 2101 g) showed craniofacial abnormalities postnatally, which suggested diagnosis of Goldenhar syndrome. Transthoracic echocardiography revealed abnormal drainage of the left pulmonary veins into the innominate vein, a large atrial septal defect, a small ventricular septal defect, and patent ductus arteriosus. Computed tomography confirmed right lung agenesis (Fig. 1A–D). Transthoracic echocardiography performed 2 days after birth revealed accelerated pulmonary venous flow between the vertical and the innominate vein. Progressive pulmonary venous obstruction necessitated surgical repair 3 days after birth.

**Fig. 2:**
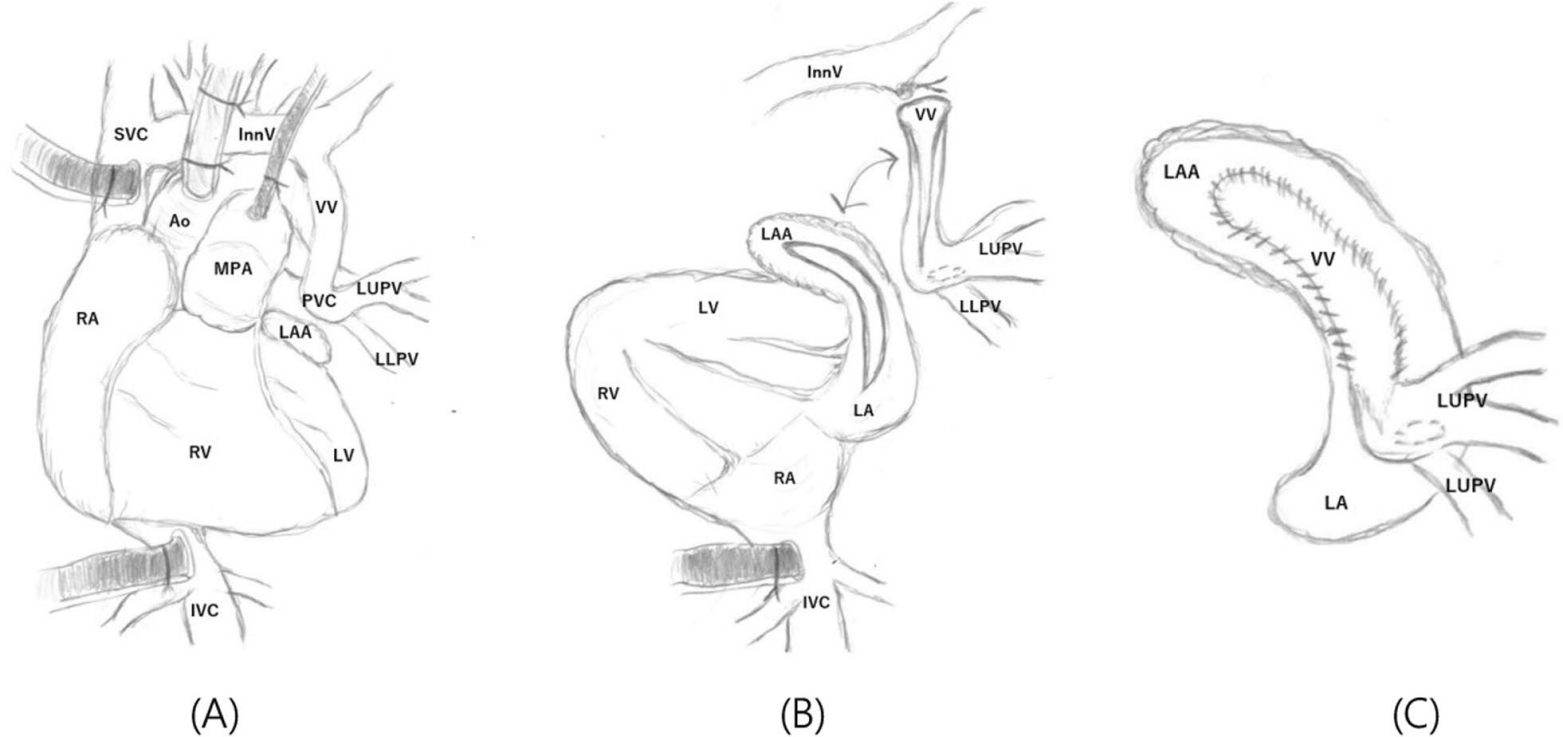
Surgical repair of the total anomalous pulmonary venous connection. **A** Cardiopulmonary bypass was established with ascending aortic perfusion and direct bicaval venous drainage. **B** The vertical vein was ligated at its confluence with the innominate vein and was divided and incised toward the pulmonary venous chamber. The corresponding posterior wall of the left atrium was incised longitudinally toward the left atrial appendage. Between the pulmonary venous chamber with vertical vein and the left atrium was anastomosed using 7–0 PDS running sutures. **C** The pulmonary venous chamber containing the vertical vein was anastomosed to the left atrium using 7–0 PDS running sutures. LUPV, left pulmonary vein; LLPV, left lower pulmonary vein; MPA, main pulmonary artery; VV, vertical vein; InnV, innominate vein; SVC, supra vena cava; Ao, aorta; IVC, inferior vena cava; RA, right atrium; LA, left atrium; RV, right ventricle; LV, left ventricle; LAA, left atrial appendage; PVC, pulmonary venous chamber

**Fig. 3:**
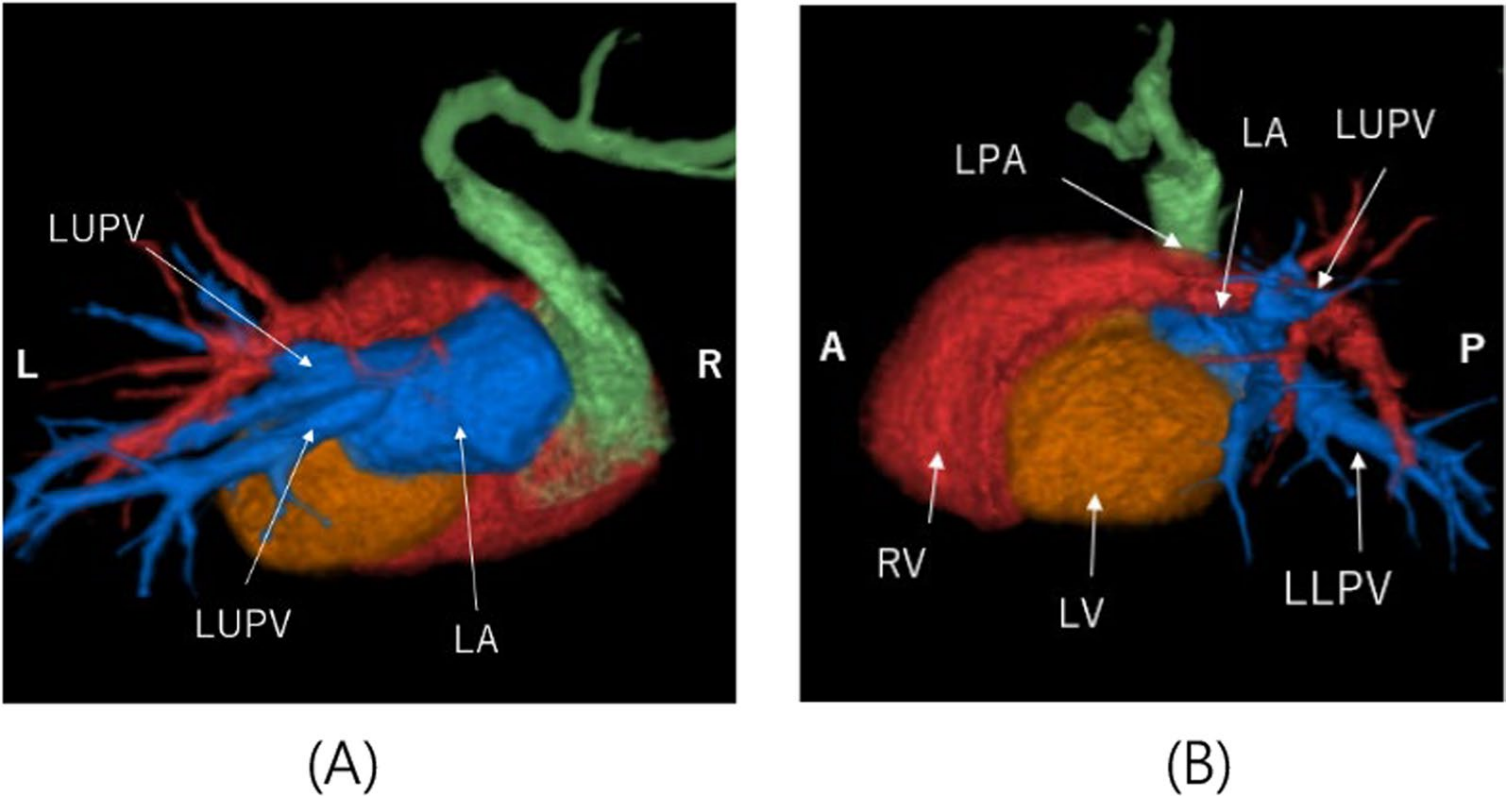
Postoperative 3-dimensional computed tomography. **A**, **B** The left pulmonary veins connected to the left atrium (posterior to anterior view and left side to right side view). LUPV, left pulmonary vein; LLPV, left lower pulmonary vein; LPA, left pulmonary artery; VV, vertical vein; InnV, innominate vein; RV, right ventricle; LV, left ventricle; LA, left atrium; LPA, left pulmonary artery

A median sternotomy was performed to expose the heart, which was rotated counterclockwise owing to right lung agenesis. Cardiopulmonary bypass was established with ascending aortic perfusion and direct bicaval venous drainage. The patent ductus arteriosus was ligated. We performed pulmonary artery venting via the left pulmonary artery, and cardiac arrest was achieved using antegrade cardioplegia infusion. The pulmonary venous chamber was well visualized after counterclockwise rotation of the heart, and we selected the posterior approach intraoperatively. The vertical vein was ligated at its confluence with the innominate vein and was divided and incised toward the pulmonary venous chamber. The corresponding posterior wall of the left atrium was incised longitudinally toward the left atrial appendage. The pulmonary venous chamber containing the vertical vein was anastomosed to the left atrium using 7–0 PDS running sutures (Fig. 2A–C). The atrial septal defect was directly closed. The aortic clamp time was 48 min. The left and right ventricular function was good with catecholamine support and nitric oxide administration. The heart was weaned off cardiopulmonary bypass at a systemic pressure of 58/35 mmHg, pulmonary pressure of 34/13 mmHg, and central venous pressure of 6 mmHg, followed by sternal closure. Echocardiography and computed tomography confirmed absence of pulmonary venous obstruction, 1 year postoperatively (Fig. 3A, B).

## Discussion

Goldenhar syndrome is a rare congenital disease that has been reported in patients with various cardiac and lung malformations. The most frequent congenital abnormality observed in patients with Goldenhar syndrome is tetralogy of Fallot, followed by septal defects and situs inversus. Total anomalous pulmonary venous connection concomitant with lung agenesis is reported in only 6 cases in the available literature [2–4] and total anomalous pulmonary venous connection accompanied by unilateral lung agenesis and Goldenhar syndrome in only 2 cases; however, surgical outcomes remain unknown in these cases [3, 4]. To our knowledge, this is the first report that describes a patient who has survived over 1 year postoperatively.

A median sternotomy or posterolateral thoracotomy is the usual surgical approach selected in such cases.

Hasegawa et al. [4] reported that median sternotomy was associated with technical difficulties in patients with right unilateral pulmonary agenesis because of rotation and displacement of the heart into the right hemithorax. Innominate vein cannulation is performed for cardiopulmonary bypass because superior vena cava and right atrial access is difficult in patients with right unilateral pulmonary agenesis, who undergo median sternotomy. Venous cannulation is relatively easy in patients with right unilateral pulmonary agenesis, who undergo right posterolateral thoracotomy.

Pietrzykowski et al. [5] observed that venous cannulation was challenging via a median sternotomy to gain access to structures that were more posteriorly positioned than usual. In our view, a median sternotomy scored over posterolateral thoracotomy to anastomose the pulmonary venous chamber and the left atrium in our patient. Therefore, we selected a median sternotomy approach. The bicaval venous cannula was easily inserted, and the pulmonary venous chamber and posterior wall of the left atrium were well visualized via the posterior approach.

A study has reported the efficacy of a sutureless approach for surgical management of postoperative pulmonary vein stenosis, following total anomalous pulmonary venous connection [6]. However, a sutureless approach was deemed unsuitable in our patient owing to the small size of the pulmonary venous chamber. Therefore, we enlarged the pulmonary venous chamber using the vertical vein and anastomosed it to the left atrium. No stenosis was observed between the pulmonary venous chamber and the left atrium at the 1-year postoperative follow-up.

## Conclusion

We report a rare case of total anomalous pulmonary venous connection accompanied by unilateral lung agenesis and Goldenhar syndrome, which was successfully repaired 3 days after birth. A median sternotomy is a safe and effective approach in patients with congenital heart disease accompanied by unilateral lung agenesis. Repair of the supra cardiac total anomalous pulmonary connection using the vertical vein is feasible in patients with a small pulmonary venous chamber.

## Data Availability

The data that support the findings of this report are available from Chiba Children’s Hospital. The author can make it available upon reasonable request.

## Abbreviations

LPV: Left pulmonary vein
LUPV: Left upper pulmonary vein
LLPV: Left lower pulmonary vein
LPA: Left pulmonary artery
VV: Vertical vein
InnV: Innominate vein
SVC: Supra vena cava
Ao: Aorta
IVC: Inferior vena
RA: Right atrium
LA: Left atrium
RV: Right ventricle
LV: Left ventricle
LAA: Left atrial appendage
PVC: Pulmonary venous chamber
